# Supplementary material for: Quantum Chemistry Study on the Structures and Electronic Properties of Bimetallic Ca2-Doped Magnesium Ca2Mgn (n = 1–15) Clusters
Source: Nanomaterials (Basel). 2022 May 12;12(10):1654. doi: 10.3390/nano12101654 (PMC9144718; doi:10.3390/nano12101654)
Supplement: Supplementary file 1 [file nanomaterials-12-01654-s001.zip › nanomaterials-1710117-supplementary.pdf]

nanomaterials

Supporting Information

Quantum Chemistry Study on the Structures and Electronic Properties of Bimetallic Ca_2-Doped Magnesium Ca_2Mg_n (n = 1–15) Clusters

Chenggang Li [1,2], Yingqi Cui [1,*], Hao Tian [1], Baozeng Ren [2], Qingyang Li [3], Yuanyuan Li [3] and Hang Yang [3]

1 Quantum Materials Research Center, College of Physics and Electronic Engineering, Zhengzhou Normal University, Zhengzhou 450044, China; zznu_lcg@163.com (C.L.); zznu_lf@163.com (H.T.)
2 School of Chemical Engineering and Energy, Zhengzhou University, Zhengzhou 450001, China; zznurbz@163.com
3 School of Physics and Electronic Engineering, Sichuan University of Science & Engineering, Zigong 643000, China; zznmuszg@163.com (Q.L.); zznucyq@163.com (Y.L.); zznutyn@163.com (H.Y.)
* Correspondence: zznu_llm@163.com

Table S1. Cartesian coordinates for the lowest energy structures of Ca_2Mg_n (n = 1–15) clusters.

Ca_2Mg
0 1

Ca	0.00000000	1.97058300	−0.68205300
Ca	0.00000000	−1.97058300	−0.68205300
Mg	0.00000000	0.00000000	2.27350900

Ca_2Mg_2
0 1

Ca	0.00000000	1.88425800	−0.88419300
Ca	0.00000000	−1.88425800	−0.88419300
Mg	−1.51752100	0.00000000	1.47365600
Mg	1.51752100	0.00000000	1.47365600

Ca_2Mg_3
0 1

Ca	0.00000000	1.83263400	−0.81324900
Ca	0.00000000	−1.83263400	−0.81324900
Mg	2.81872900	0.00000000	0.39575300
Mg	−2.81872900	0.00000000	0.39575300
Mg	0.00000000	0.00000000	1.91932500

Ca_2Mg_4
0 1

Ca	−1.78885400	0.00000000	−0.52349800
Ca	1.78885400	0.00000000	−0.52349800
Mg	0.00000000	2.94728100	−0.97558700
Mg	0.00000000	1.70811500	1.84808400
Mg	0.00000000	−1.70811500	1.84808400
Mg	0.00000000	−2.94728100	−0.97558700

Ca_2Mg_5
0 1

Ca	0.00000000	0.00000000	1.81816700
Ca	0.00000000	0.00000000	−1.81816700
Mg	0.00000000	2.76593700	0.00000000
Mg	−2.63056200	0.85472100	0.00000000
Mg	−1.62577700	−2.23769000	0.00000000
Mg	1.62577700	−2.23769000	0.00000000
Mg	2.63056200	0.85472100	0.00000000

Ca_2Mg_6
0 1

Ca	0.16527200	−0.41441400	1.47367600
Ca	−2.04519400	1.59424500	−0.67154700
Mg	−1.24113100	−1.67839400	−1.27475800
Mg	−3.08563200	−1.21126600	1.17819600
Mg	1.10875000	0.42864300	−1.59597300
Mg	1.91239200	−2.44275500	−0.61957900
Mg	3.27738800	0.24216700	0.55770500
Mg	1.16143700	2.69522000	0.41752900

Ca_2Mg_7
0 1

Ca	0.00000000	3.00348500	−1.47713500
Ca	0.00000000	−3.00348500	−1.47713500
Mg	−1.52724200	−1.63782500	1.19458800
Mg	1.52724200	1.63782500	1.19458800
Mg	−1.52848800	0.00000000	−1.56010600
Mg	0.00000000	0.00000000	3.26564300
Mg	−1.52724200	1.63782500	1.19458800
Mg	1.52848800	0.00000000	−1.56010600
Mg	1.52724200	−1.63782500	1.19458800

Ca_2Mg_8
0 1

Ca	−3.19321400	−0.58554500	0.00000000
Ca	0.07323100	3.60247500	0.00000000
Mg	−0.64076900	0.85158700	1.59367300
Mg	2.06483700	−2.09803900	0.00000000
Mg	−0.64076900	0.85158700	−1.59367300
Mg	1.78960800	−0.62248800	2.60427200
Mg	−0.64076900	−2.17382400	1.53186700
Mg	1.78960800	−0.62248800	−2.60427200
Mg	2.11899400	0.95927200	0.00000000
Mg	−0.64076900	−2.17382400	−1.53186700

Ca_2Mg_9
0 1

Ca	2.18820300	−1.73718700	0.94349000
Ca	−2.18820300	1.73718700	0.94349000
Mg	0.00000000	4.18801500	−0.32866600
Mg	1.22298000	1.49958700	0.81206200
Mg	0.00000000	−4.18801500	−0.32866600
Mg	−2.56555800	−0.48263200	−1.65266600
Mg	−1.22298000	−1.49958700	0.81206200
Mg	0.33804200	−1.51365900	−1.92409000
Mg	−0.33804200	1.51365900	−1.92409000
Mg	0.00000000	0.00000000	3.04175400
Mg	2.56555800	0.48263200	−1.65266600

Ca_2Mg_{10}
0 1

Ca	2.45105000	−0.56590800	−2.16179900
Ca	−2.04815100	−0.35884000	1.72433100
Mg	−0.74406900	−0.17590300	−1.51768900
Mg	−2.34796300	2.27743100	−0.48737800
Mg	−2.30350300	−2.59634100	−0.66196300
Mg	0.55364200	−2.21946900	0.26631600
Mg	1.19439100	−0.24137700	2.72193700
Mg	0.01902800	2.17867200	1.43588400
Mg	3.40873500	−1.40910400	0.87494300
Mg	2.89815400	1.50001700	0.55832200
Mg	0.74755900	2.34892300	−1.48450500
Mg	−4.09747200	−0.12160300	−0.97675300

Ca_2Mg_{11}
0 1

Ca	−2.57338600	−2.44918400	0.00000000
Ca	2.49307100	−1.83285700	0.00000000
Mg	2.11494200	1.19348800	1.52146000
Mg	−2.23703900	0.96484800	0.00000000
Mg	0.14879600	−3.78647900	−1.46705500
Mg	−0.53460100	2.36711800	−2.25735800
Mg	−1.72472700	4.01574400	0.00000000
Mg	1.21885700	3.68531700	0.00000000
Mg	−0.29075200	−0.53871400	−1.66059100
Mg	−0.53460100	2.36711800	2.25735800
Mg	0.14879600	−3.78647900	1.46705500
Mg	2.11494200	1.19348800	−1.52146000
Mg	−0.29075200	−0.53871400	1.66059100

Ca_2Mg_{12}
0 1

Ca	0.08493100	2.84409100	0.00000000
Ca	3.10899100	0.35267600	0.00000000
Mg	−2.06143200	−1.83578900	1.59087600
Mg	−2.70965100	0.92933000	0.00000000
Mg	−1.68374900	1.28681100	2.82436200
Mg	1.31595900	1.00535100	2.76502500
Mg	1.50109400	−2.17851400	−1.77966500
Mg	−0.38225900	−1.24187800	−3.97785200
Mg	−1.68374900	1.28681100	−2.82436200
Mg	0.00721900	−0.32923600	0.00000000
Mg	1.31595900	1.00535100	−2.76502500
Mg	−0.38225900	−1.24187800	3.97785200
Mg	1.50109400	−2.17851400	1.77966500
Mg	−2.06143200	−1.83578900	−1.59087600

Ca_2Mg_{13}
0 1

Ca	−1.11784600	0.01333400	2.84620500
Ca	−1.11784600	0.01333400	−2.84620500
Mg	−1.60318700	1.88107000	0.00000000
Mg	1.73863500	−1.83466600	−2.42811700
Mg	−0.49977300	3.31499100	−2.43218200
Mg	1.73863500	−1.83466600	2.42811700
Mg	−0.92630700	−3.20367200	−1.59046900
Mg	−0.49977300	3.31499100	2.43218200
Mg	1.71555300	−3.60551900	0.00000000
Mg	1.19712000	3.85224400	0.00000000
Mg	−0.92630700	−3.20367200	1.59046900
Mg	1.86939300	1.50835400	−1.84626500
Mg	−2.51867700	−1.21464300	0.00000000
Mg	1.86939300	1.50835400	1.84626500
Mg	0.57145100	−0.52761500	0.00000000

Ca_2Mg_{14}
0 1

Ca	1.44381900	−0.16949800	2.78990400
Ca	1.44381900	−0.16949800	−2.78990400
Mg	−0.13387400	−0.17565200	0.00000000
Mg	2.63381200	−1.92822900	0.00000000
Mg	0.50809400	−3.25289100	1.55898000
Mg	−2.12287300	−3.15402300	0.00000000
Mg	0.50809400	−3.25289100	−1.55898000
Mg	−1.73185400	−1.52746900	−2.39772300
Mg	−1.73185400	−1.52746900	2.39772300
Mg	−1.61701100	3.46355100	0.00000000
Mg	−1.41203300	1.80823800	2.41775400
Mg	2.66883600	1.31175500	0.00000000
Mg	−1.41203300	1.80823800	−2.41775400
Mg	0.99718600	3.17082600	1.53967200
Mg	0.99718600	3.17082600	−1.53967200
Mg	−2.96440900	0.65018100	0.00000000

Ca_2Mg_{15}
0 1

Ca	0.00000000	0.00000000	0.30469300
Ca	0.00000000	0.00000000	3.71622200
Mg	1.47949500	−2.63392400	1.65915500
Mg	1.49551000	−0.86343300	−3.36982300
Mg	−1.49551000	−0.86343300	−3.36982300
Mg	−3.02079200	0.03568200	1.65915500
Mg	1.54129800	2.59824200	1.65915500
Mg	2.56902100	1.48322500	−1.29964900
Mg	−1.47949500	−2.63392400	1.65915500
Mg	0.00000000	1.72686700	−3.36982300
Mg	0.00000000	−2.96645000	−1.29964900
Mg	−2.56902100	1.48322500	−1.29964900
Mg	−1.54129800	2.59824200	1.65915500
Mg	0.00000000	3.30884600	−0.88268100
Mg	3.02079200	0.03568200	1.65915500
Mg	2.86554500	−1.65442300	−0.88268100
Mg	−2.86554500	−1.65442300	−0.88268100
